# KLF14 activates the JNK-signaling pathway to induce S-phase arrest in cervical cancer cells

**DOI:** 10.3389/fimmu.2023.1267950

**Published:** 2023-12-07

**Authors:** Ying Du, Hui Ye, Mei Lin, Lili Cao

**Affiliations:** ^1^ Oncology Department, Shandong Provincial Qianfoshan Hospital, School of Medicine, Shandong University, Jinan, China; ^2^ Department of Pathology, School of Basic Medicine, Qingdao University, Qingdao, China; ^3^ Oncology Department, The First Affiliated Hospital of Shandong First Medical University & Shandong Provincial Qianfoshan Hospital, , Jinan, China

**Keywords:** cervical cancer, KLF14, cell cycle, MAPK-signaling pathway, CDK2

## Abstract

**Objective:**

To explore the role of Krüppel-like factor 14 (KLF14) and its underlying mechanism(s) of action in cell-cycle regulation in cervical cancer.

**Methods:**

Lentiviral infection was used to construct *KLF14*, *KLF14* zinc-finger structural mutations, and empty vector controls in SiHa and HeLa cervical cancer cells. The effect of KLF14 on cervical cancer cell cycle was detected by flow cytometry. The effect of KLF14 on the expression of cyclin-dependent kinase 2 (*CDK2*), cyclin A2 (CCNA2*)*, and MAPK signalling pathway-related molecules was detected by fluorescence quantitative RT-PCR (qRT-PCR) and western blot. Cervical cancer cells were treated with JNK-pathway inhibitors/agonists before we assessed changes in the cell cycle and the expression of the CDK2, CCNA2, and p-JNK/JNK. Subcutaneous xenograft studies to explore the effects of KLF14 on cervical cancer cell proliferation *in vivo*, and western blotting was implemented to measure the expression of CCNA2, CDK2, and the activation levels of the MAPK-signaling pathway proteins in tumours.

**Results:**

The proportion of cells in the S phase was increased in the *KLF14*-overexpressing group compared with the control group (*P*<0.001); CDK2, CCNA2, and *p*-JNK/JNK expression levels were elevated in the *KLF14-*overexpressing group relative to the control group (all *P*<0.05). When JNK-pathway activation was inhibited/promoted, the proportion of cells in the S phase was reduced/increased (*P*<0.05) and CDK2 and CCNA2 expression levels were reduced/decreased, respectively (all *P*<0.05). Vivo experiments revealed that KLF14 inhibited cervical cancer cell proliferation (*P*<0.01) and that *p*-JNK/JNK, CDK2, and CCNA2 expression levels were augmented in tumours in the overexpression group (*P*<0.01).

**Conclusion:**

KLF14 induced S-phase arrest in cervical cancer cells and inhibited the proliferation of cervical cancer cells *in vivo*; the induction of S-phase arrest was related to its zinc-finger structure. KLF14 also activated the JNK pathway to induce S-phase arrest and promote the expression of CDK2 and CCNA2. In summary, KLF14 activates the JNK-signaling pathway to induce S-phase arrest in cervical cancer cells

## 1 Introduction

Cervical cancer (CC) is the second leading cause of cancer deaths in young women worldwide (1). According to the GLOBOCAN 2020 database, the incidence rate of cervical cancer is 13.3 per 100,000, and the mortality rate is 7.3 per 100,000. The incidence and mortality rates of cervical cancer have significantly increased in recent years (2). Even though various preventive and treatment measures have emerged, surgery is still the first choice for cervical cancer treatment. Most patients are already in the advanced clinical stage when diagnosed, beyond the optimal period of surgery, and need to rely on chemotherapy. However, with the development of chemotherapy resistance, the therapeutic effect of cervical cancer is affected. The global cervical cancer burden remains high (3). Previous studies have shown that the reentry cell cycle of differentiated cells is one of the main reasons affecting the occurrence, development, and drug resistance of cervical cancer (4–8). It’s necessary to explore the mechanism of inhibiting the reentry cell cycle of cervical cancer cells. To regulate the cell cycle and provide targets for anti-cancer therapy (9, 10).

Krüppel-like factor 14 (*KLF14*), a member of the Krüppel-like transcription factor family (KLFs) which can activate or inhibit genes involved in cell cycle regulation to further regulate the cell cycle, acting as a key regulator of tumor pathogenesis (11). Initial studies on *KLF14* focused on lipid and glucose metabolism (12). Recent studies revealed that *KLF14* was downregulated in colorectal cancer, breast cancer, lymphoma, cervical cancer, cancer of the floor of the mouth, and pancreatic cancer (13–18). *KLF14* can inhibit cancer in colorectal cancer, breast cancer, hepatocellular carcinoma, and other tumors (13, 15–17, 19). *KLF14* inhibition caused centriole amplification, aneuploidy, and spontaneous tumorigenesis—while forced expression of *KLF14* generated mitotic abnormalities and increased DNA damage (20). Previous studies of my research group found that *KLF14* targets ITGB1, promotes apoptosis through the PI3K/AKT signaling pathway, and inhibits the progression of cervical cancer (18). However, the effect and mechanism of *KLF14* on the cervical cancer cell cycle have not been unclear.

The mitogen-activated protein kinase (MAPK) signaling pathway is a crucial signal transduction pathway in cells, and its members include c-Jun N-terminal kinase (JNK), extracellular signal-regulated kinase (ERK), and p38 MAPK. MAPK signaling pathway participating in many important life processes such as cell cycle, proliferation, apoptosis, migration, and invasion. The occurrence and development of obesity, diabetes, cancer, and other diseases are closely related to the activation of this pathway (21–23). Research showed that *KLF14* relies on MAPK signaling pathways to increase oxidative adaptation in castration-resistant prostate cancer (24). *KLF14* may affect the expression of cellular inflammatory factors through the MAPK signaling pathway and play an important role in the middle stage of atherosclerotic lesion formation (25). At present, the relationship between *KLF14* and MAPK signaling pathway is rarely studied in tumors. As a key kinase regulating to entry and progression of the S phase, CDK2 mainly binds to the cell cycle protein CyclinA2 and plays a role. In multiple myeloma, cervical cancer, and hepatocellular carcinoma, the MAPK signaling pathway is closely associated with CDK2 and cyclin A2 (26–28). Therefore, we considered whether KLF14, CDK2, CyclinA2 and MAPK signaling pathways are correlated to affect the cell cycle of cervical cancer.

In this study, we investigated whether KLF14 can inhibit the proliferation and induce S-phase arrest of cervical cancer cells. Moreover, we further explored whether KLF14 regulates CDK2 and CyclinA2, and plays an inhibitory role in the progression of cervical cancer by regulating the JNK/MAPK signaling pathway.

## 2 Materials and methods

### 2.1 Cell culture

Human cervical cancer cells, including SiHa, HeLa, C33a, and Caski cells, were purchased from the Chinese Tissue Culture Collections (CTCC, China) and the American Type Culture Collection (ATCC, USA). These cells were cultured in DMEM (Gibco, USA) containing 10% fetal bovine serum (FBS, Gibco, USA) and 1% penicillin/streptomycin mix and incubated in a constant-temperature incubator at 37°C with 5% CO2.

### 2.2 Cell transfection

The KLF14 mRNA expression level of cervical cancer cells was detected by qRT-PCR. SiHa and HeLa cells with low endogenous KLF14 expression were selected for overexpression.

KLF14 overexpression lentivirus (OE-KLF14) and its negative control lentivirus (OE-Ctrl) were constructed by GeneChem (Shanghai, China). OE-KLF14 and OE-Ctrl were infected into SiHa or HeLa cells.

**Figure 1:**
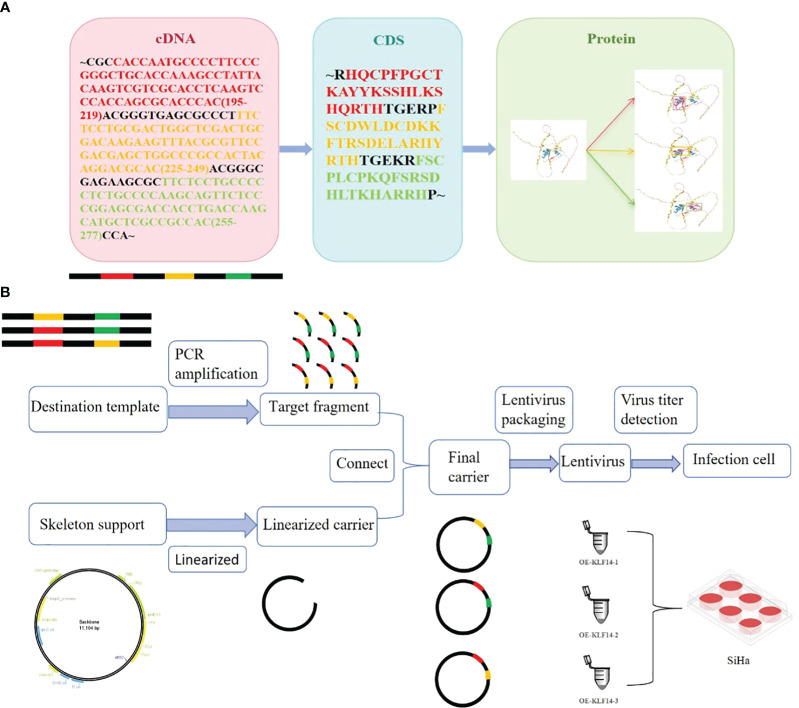
Construction of mutant protein of zinc finger structure of transcription factor KLF14. **(A)** KLF14 protein contains three highly conserved zinc finger structures, and their corresponding cDNA sequences and CDS sequences were queried from NCBI. **(B)** The cDNA sequence corresponding to the structure of the first, second, or third zinc finger is deleted by gene editing technology, and the edited target fragment is amplified by PCR; The skeleton vector pHS-AVC-1403 was linearized and connected with the amplified target fragment to construct KLF14 zinc finger mutant plasmid. The constructed final plasmid was packaged into the KLF14 zinc finger mutant lentiviruses OE-KLF14-1, OE-KLF14-2, and OE-KLF14-3. SiHa cells were infected with lentivirus and the mutant protein of KLF14 zinc finger structure was stably expressed in the cells.

Construct KLF14 zinc finger mutant lentivirus. The transcription factor KLF14 contains three zinc finger structures. The CDS sequence of KLF14 and the amino acid sequence corresponding to the structure of three zinc fingers were retrieved from NCBI (**Figure 1A**). By using gene editing technology to delete specific base sequences to obtain base sequences that retain specific zinc finger structures, which is the target sequence. For example, when OE-KLF14-1 lentivirus was constructed, the base sequences corresponding to the first zinc finger structure were deleted, while the base sequences corresponding to the second and third zinc finger structures were retained. PCR was used to amplify the target fragment. The skeleton vector pHS-AVC-1403(PLV-TRE-MCS-3FLAG-HEF1A-RITTA-P2A-PURO) was used to construct the KLF14 zinc-finger mutant plasmids. The amplified target fragment and skeleton carrier were digested with the same restriction enzyme EcoRI. After the digestion was completed, the respective target bands were recovered by agar-gel electrophoresis. After recovering the target bands to obtain the recovered products, T4 ligase was used to connect the recovered products of the target fragment and skeleton carrier. Construct KLF14 zinc finger mutant plasmid. After plasmid amplification, electrophoresis quality control, lentivirus packaging, and quality testing, KLF14 zinc finger mutant lentivirus was finally obtained (**Figure 1B**). The construction of mutant protein of zinc finger structure of transcription factor KLF14 can be seen in the Chinese patent applied by the author (patent number: CN116514947A). KLF14 zinc finger mutant lentivirus (OE-KLF14-1, OE-KLF14-2, OE-KLF14-3) was purchased from Beijing Hopson Biotechnology Co., Ltd. SiHa cells were infected with KLF14 zinc finger structure mutant lentivirus (**Figure 1B**). Stable cell clones were obtained after screening with 5μg/ml puromycin.

### 2.3 Validation of infection efficiency

Cells were cultured with complete medium with doxorubicin (DOX, 5μg/ml, CST, #HY-N0565B) for 24h which can induce KLF14 expression in cervical cancer cells and the infection efficiency was verified by qRT-PCR and Western blotting.

The mRNA expression of KLF14 in the OE-KLF14 group and control group was detected by qRT-PCR. Total RNA was extracted from stably transfected cells with total RNA rapid extraction kit (Feijie, China). Total RNA (1 μg) was reverse-transcribed into first-strand complementary DNA using a PrimeScript^®^ RT Reagent Kit (TaKaRa, Japan). PCR was performed with a SYBR Green PCR Kit (TaKaRa, Japan) using Bio-Rad CFX96. Primers for KLF14 (Sangon Biotech, China) were listed as follows, KLF14 Forward: 5′-TTCATCCAGGGGAGGTACAAC-3′; KLF14 Reverse: 5′-CCAGGAGTTACTTCTATGCCTGA-3′; β-actin Forward: 5′-AGTTGCGTTACACCCTTTC-3′, β-actin Reverse: 5′-CCTTCACCGTTCCAGTTT-3′. All samples were normalized to β-actin levels. Gene-specific relative mRNA levels were calculated by the standard equation 2^-(ΔCT sample-ΔCT control)^.

The protein expressions of Flag and KLF14 in the OE-KLF14 group and control group were detected by Western blotting.

The total cell protein was exacted with RIPA and sample buffer solution (Beyotime,China). Equal amounts of proteins were separated by 12.5% SDS-PAGE (Epizyme, China) and transferred onto 0.45 μM PVDF membranes (Millipore, America). After being blocked with 5% skim milk power for 1 h and washed three times by TBST. The membranes were incubated with the primary antibodies KLF14 (1:1000, #PA5-23784, Thermo Fisher), Flag (1:1000, #14793, CST), and β-Actin antibody (1:1000, BM0627,Bode Biological Engineering Co, LTD) at 4°C overnight.

The membranes were washed three times with TBST. Membranes were incubated with suitable secondary antibodies goat anti−mouse IgG (1:3000, 7076S, CST) or goat anti−rabbit IgG (1:3000, 7074S, CST) at room temperature for 1 h. The membranes were washed three times with TBST.

ECL chemiluminescence (Millipore, America) was utilized to detect the blots. Protein levels were normalized to β-Actin. ImageJ was applied to measure the gray value of the strip. Relative protein expression = (the experimental group’s target protein/the experimental group’s β-Actin)/(the control group’s target protein/the control group’s β-Actin).

### 2.4 Cell cycle detection

We examined the cell cycles of cervical cancer cells in the groups designated as OE-Ctrl, OE-KLF14-1, OE-KLF14-2, OE-KLF14-3, and OE-KLF14. Cervical cancer cells in the OE-Ctrl and OE-KLF14 groups were left untreated or treated with SP600125 (#HY-12041, MCE)/anisomycin (#HY-18982, MCE) to measure the effects of JNK-pathway activation levels on cell cycle. We used trypsin to digest the cells and washed them with pre-cooled PBS twice. Cells were counted using the Countstar Automated Cell Counter (Ruiyu, Shanghai) and the cell concentration was adjusted to 1×10^6^ cells/ml. We added one milliliter of cellular suspension to each 1.5-ml EP tube, and after centrifugation the supernatant was discarded and 500 μl of pre-cooled 70% ethanol was added. After fixation at 4°C for 4 h, the cells were centrifuged and the supernatant was again discarded. Cells were then resuspended with 500 μl of pre-cooled PBS, centrifuged, and the supernatant was discarded. The cells were re-suspended with 100 μl of RNase (Solarbio, China) and incubated in water at 37°C for 30 min. All cells were treated with 400 μl of PI staining solution (Solarbio, China) at 4°C in the dark for 30 min. The cells were passed through a 200-mesh filter before flow cytometry (Agilent, China) was implemented to analyze the cell cycle of the various groups.

### 2.5 CDK2 mRNA expression

We applied qRT-PCR to measure *CDK2* mRNA expression levels of the OE-Ctrl and OE-KLF14 groups. Cervical cancer cells in the OE-Ctrl and OE-KLF14 groups were treated with or without SP600125/anisomycin to determine the effects of JNK-pathway activation levels on *CDK2* expression (the methods used were the same as 1.3). Primers for CDK2 were listed as follows: CDK2 Forward: 5′-TTCATCCAGGGGAGGTACAAC-3′; CDK2 Reverse: 5′-CCAGGAGTTACTTCTATGCCTGA-3′.

### 2.6 Expression of CDK2, CyclinA2 and MAPK pathways

We used western blotting to evaluate the expression of CDK2, CyclinA2, and MAPK pathway related molecules in the OE-Ctrl and OE-KLF14 groups. Primary antibodies P38(1:1000, #8690), p-P38(1:1000, #4511), JNK(1:1000, #9252), p-JNK(1:1000, #4668), ERK(1:1000, #4695), p-ERK(1:1000, #4370), CDK2(1:1000, #18048) were purchased from CST, USA. Primary antibodie CyclinA2 (1:1000, #ab181591) was purchased from Abcam. We treated cervical cancer cells with or without SP600125/anisomycin to measure the effects of JNK-pathway activation levels on CDK2 and CyclinA2 expression. The methods used were the same as 1.3.

### 2.7 Xenograft assay *in vivo*

Female 4-week-old BALB/c (nu/nu) nude mice were purchased from Vital River Lab Animal Technology Co., Ltd. (Beijing, China). 5×10^6^ cells suspended 200 μl PBS were injected subcutaneously in to mice (n=8). SiHa cells of the OE-KLF14 group and OE-Ctrl group were injected subcutaneously into the right and left axilla of nude mice, respectively. During the modeling period, subcutaneous tumour growth was observed every 5 days. The tumour volume was calculated using the formula V = (larger diameter) × (smaller diameter)^2^/2. The growth curve of tumour volume was drawn. Approximately forty days later, the nude mice were euthanized under anesthesia. The tumours had formed under the skin were removed, photographed, weighed, and frozen at -80°C. The tumour tissue proteins of the OE-control group and OE-KLF14 group were extracted by JXFSTPRP-24 tissue homogenizer (Jingxin, Shanghai) for Western blotting, and the expression of KLF14, CDK2, CyclinA2, and MAPK pathway related molecules in tumour tissues was detected. This experiment was approved by the Ethics Committee of the First Affiliated Hospital of Shandong First Medical University (SYDWLS2020016), animal experiments strictly complied with national laws, regulations, and standards related to experimental animals, including the Regulations on the Management of Experimental Animals and the Guidelines for Ethical Review of Experimental Animal Welfare, and referred to the consensus of relevant guidelines on animal experimental research reports in international biomedical journals (ARRIVE Guidelines).

### 2.8 Statistical processing

All experiments were analyzed with GraphPad Prism 8.0.1 and SPSS 25.0. The *Shapiro-Wilk*(*S-W*) test was used to assess whether the data were normal distributed. The normal distribution was represented by *Mean ± SEM*. Independent sample t-test was used for comparison between two independent samples, paired sample t-test was used for comparison between paired samples, and one-way ANOVA was used for comparison in more than two groups. For data that did not conform to normal distribution, the *Mann-Whitney* test was used for comparison between two independent samples, the Wilcoxon rank sum test was used for the comparison between paired samples, and the *Kruskal-Wallis* test was used for comparison in multiple independent samples. *a*=0.05 was used as the test level, and *P* < 0.05 was considered statistically significant.

## 3 Results

### 3.1 Construct cervical cancer cell lines that stably overexpressed *KLF14*

**Figure 2:**
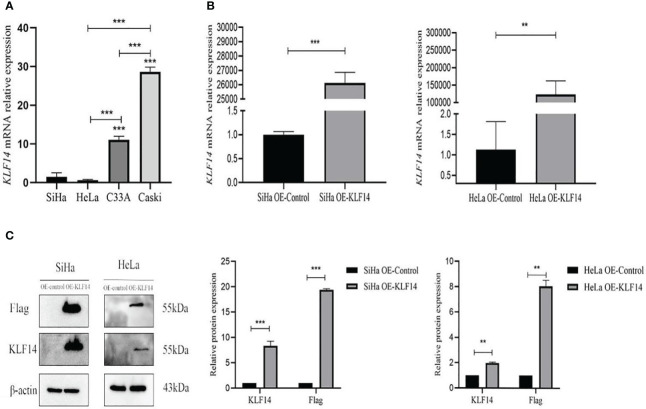
Construct CC cell lines that stably overexpressed KLF14. **(A)** KLF14 mRNA expression level of wild-type CC cells by qRT-PCR assay. **(B, C)** The transfection efficiency of KLF14 overexpressed lentivirus in SiHa and HeLa cells was detected by qRT-PCR and Western blotting (**P < 0.01, ***P < 0.001).

The *KLF14* mRNA expression levels in SiHa, HeLa, C33A, and Caski cervical cancer cell lines were measured (1.470±0.543, 0.636±0.085, 11.053±0.46852, 28.660±0.590, respectively). As the *KLF14* expression levels in SiHa and HeLa cells were lower than in C33A and Caski cells (*P*<0.001), we selected the former two cell lines for subsequent experiments (**Figure 2A**). Compared with the OE-Ctrl group, the *KLF14* mRNA expression level of the OE-KLF14 group was elevated (*P*_SiHa_<0.001, *P*_HeLa_=0.008) (**Figure 2B**), and KLF14 protein expression levels were augmented in the latter group (*P*_SiHa_<0.001, *P*_HeLa_=0.002); the expression level of Flag protein was also increased (*P*_SiHa_<0.001, *P*_HeLa_=0.001) (**Figure 2C**). We thus successfully constructed SiHa and HeLa cell lines that stably overexpressed *KLF14*.

### 3.2 *KLF14* induces S-phase arrest

**Figure 3:**
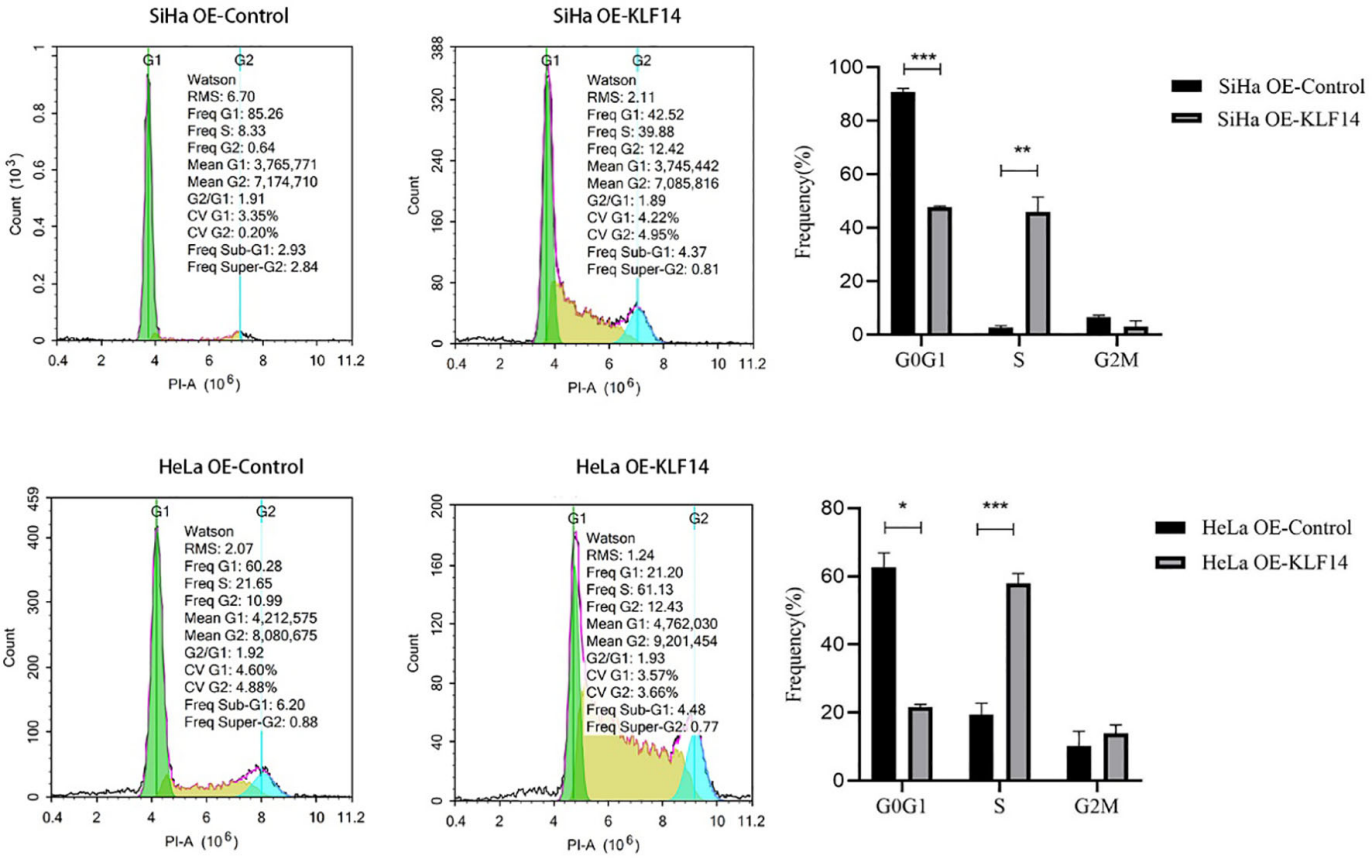
The S-phase arrest by KLF14 was observed in CC cells. SiHa and HeLa in the OE-KLF14 group and OE-Ctrl group were stained with PI dye, and DNA content was detected by flow cytometry. The cell cycle was divided according to the DNA content. (*P < 0.05, **P < 0.01, ***P < 0.001).

After *KLF14* overexpression, our statistical distribution of cell-cycle data for cervical cancer cells showed that the G0/G1 phase of the HeLa OE-Ctrl group (*P*=0.011) and the G2/M phase of the HeLa OE-KLF14 group (*P*=0.008) were not normally distributed. The ratio of cells in G0/G1 in the SiHa OE-KLF14 group was reduced relative to the control group (*P*<0.001), and the ratio of cells in the S phase was increased in the former (*P*=0.005)—with no statistically significant difference in the ratio of cells in the G2/M phase (*P*=0.058) (**Figure 3**). The ratio of cells in G0/G1 in the HeLa OE-KLF14 group was reduced relative to the control group (*P*<0.049) and the ratio of cells in the S phase was increased in the former (*P*<0.001)—with no statistically significant difference in the ratio of cells in the G2/M phase (*P*=0.275) (**Figure 3**). Thus, we observed that *KLF14* overexpression augmented the ratio of S-phase cells and reduced the ratio of the G0/G1 phase cells in cervical cancer, inducing S-phase arrest in cervical cancer cells.

### 3.3 The KLF14 zinc finger structure is essential for KLF14-induced S-phase arrest

Transcription factors achieve their biological effects by binding to promoters, and this process requires specific protein structures such as zinc fingers. KLF14 contains three zinc fingers that are closely associated with cell-cycle arrest (29).

**Figure 4:**
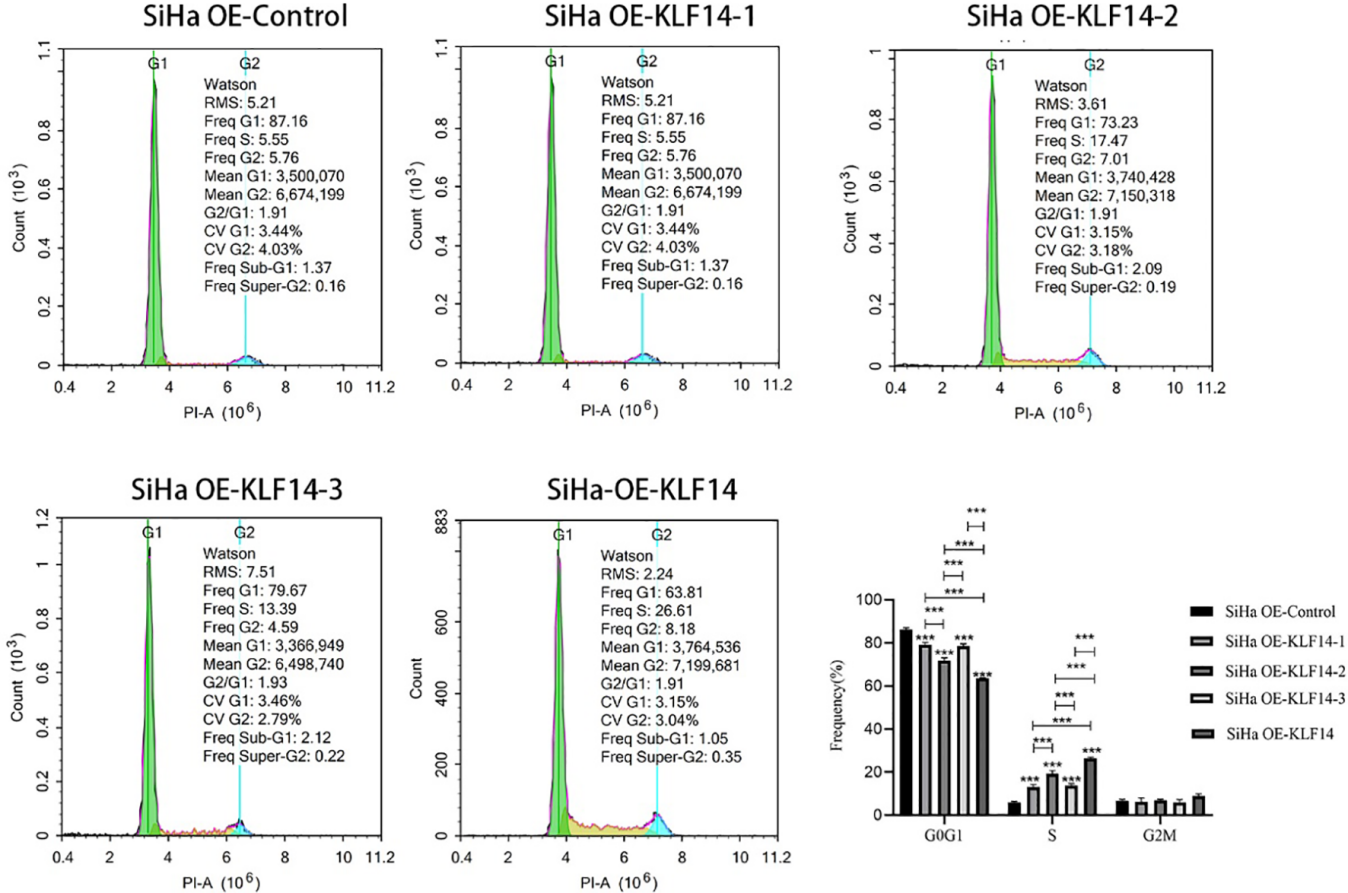
The KLF14 zinc finger structure is essential for KLF14-induced S-phase arrest. The first, second, and third zinc finger structures of KLF14 were missing respectively to construct zinc finger mutant CC cell lines: SiHa OE-KLF14-1, SiHa OE-KLF14-2, SiHa OE-KLF14-3. PI staining and flow cytometry were used to detect DNA content in mutant groups, which were compared with the SiHa OE-KLF14 and SiHa OE-Ctrl groups. (***P < 0.001).

To clarify the relationship between the KLF14 zinc finger and the cell cycle, we deleted the base sequence corresponding to the first zinc finger structure of KLF14 by gene editing technology and retained the base sequence corresponding to the second and third zinc finger structures. According to the steps in 1.2, KLF14 zinc finger mutant lentivirus OE-KLF14-1 was constructed. And so on, the lentivirus without the second zinc finger structure (OE-KLF14-2) and without the third zinc finger structure (OE-KLF14-3) was constructed. SiHa cells were infected with KLF14 zinc finger structure mutant lentivirus. SiHa cell lines OE-Ctrl, OE-KLF14-1, OE-KLF14-2, OE-KLF14-3, and OE-KLF14 groups were used for cell-cycle testing. Statistical results showed that the cell proportion of each cell cycle was normally distributed. One-way ANOVA showed that there were statistically significant differences in the ratios of cells in the G0/G1 and S phases (*P*_G0/G1_<0.001, *P_S_* <0.001) but that there was no difference in the G2/M phase (*P*_G2/M_=0.061). Pairwise comparisons of statistically significant differences revealed that the OE-KLF14-1 and OE-KLF14-3 groups did not differ concerning the G0/G1 phase; likewise, OE-KLF14-1 and OE-KLF14-3 did not differ concerning the S phase (all *P*>0.05). Pairwise comparisons of the remaining groups showed that the differences were statistically significant (*P*<0.001). KLF14-induced S-phase arrest in cervical cancer cells was therefore associated with the presence of the zinc fingers, and the loss of any zinc finger diminished cell-cycle regulation compared with function before mutation (**Figure 4**).

### 3.4 KLF14 promotes JNK-pathway activation and expression of CDK2 and CyclinA2 in cervical cancer cells.

**Figure 5:**
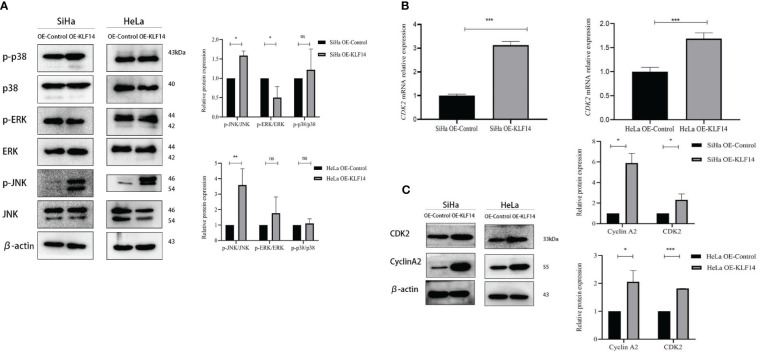
KLF14 promotes JNK-pathway activation and expression of CDK2 and CyclinA2 in CC cells. **(A)** Expression of JNK, p-JNK, ERK, p-ERK, p38, and p-p38 by Western blot assay. **(B)** Expression of CDK2 by qRT-PCR assay. **(C)** Expression of CyclinA2 and CDK2 by Western blot assay. Statistical analysis was conducted at the same time. (*P < 0.05, **P < 0.01, ***P < 0.001, ns: no significance).

The phosphorylation levels of JNK, ERK, and P38 reflect the activation status of the MAPK-signaling pathway, and our results depicted the relative expression levels of *p*-JNK/JNK, *p*-ERK/ERK, and *p*-P38/P38 proteins in the four groups as normally distributed. In the OE-KLF14 group, *p*-JNK/JNK was augmented compared to the control group (*P*_SiHa_=0.014, *P*_HeLa_=0.004), while *p*-P38/P38 did not differ (*P*_SiHa_=0.516, *P*_HeLa_=0.560). In the SiHa OE-KLF14 group, *p*-ERK/ERK was diminished compared with the control group (*P*_SiHa_=0.039). There was no difference in *p*-ERK/ERK between the HeLa OE-KLF14 group and the control group (*P*_HeLa_=0.334) (**Figure 5A**). These results showed that KLF14 promoted JNK pathway activation in cervical cancer cells.

During cell-cycle regulation, critical molecules mediate cell-cycle changes, and these occupy a vital role in cancer cell proliferation. CDK2 principally drives cells to enter the S phase and regulates S-phase progression, and CyclinA2 binds to CDK2 to perform its effects and participates in the S phase of the cell cycle. When extracted mRNA from the cells of the different groups for qRT-PCR, we observed that the expression of *CDK2* mRNA in the OE-KLF14 group was increased compared with the control group (*P*_SiHa_<0.001, *P*_HeLa_<0.001), indicating that KLF14 promoted the expression of *CDK2* mRNA in cervical cancer cells (**Figure 5B**).

The protein expression levels of CDK2 and CyclinA2 conformed to a normal distribution. CDK2 protein expression was increased in the OE-KLF14 group compared with the control group (*P*_SiHa_=0.043, *P*_HeLa_<0.001), and CyclinA2 protein expression was increased (*P*_SiHa_=0.012, *P*_HeLa_=0.015) (**Figure 5C**), showing that KLF14 promoted CDK2 and CyclinA2 protein expression in cervical cancer cells.

To further examine the correlation among KLF14-induced S-phase arrest, promotion of CDK2 and CyclinA2 expression, and activation of the JNK pathway, we adopted a highly specific JNK-phosphorylation inhibitor (SP600125) or agonist (anisomycin) to treat cervical cancer cells in the following experiments.

### 3.5 KLF14 induces S-phase arrest of cervical cancer cells and promotes the expression of CDK2 and CyclinA2 by activating the JNK-signaling pathway

Control and experimental groups (+S/+A) were set up in OE-Ctrl and OE-KLF14 groups. The control group was treated with doxorubicin (DOX) for 24 h and the experimental group was treated with the JNK-phosphorylation inhibitor (+S, 0.125 μM SP600125)/agonist (+A, 20 μM anisomycin) and DOX for 24 h.

**Figure 6:**
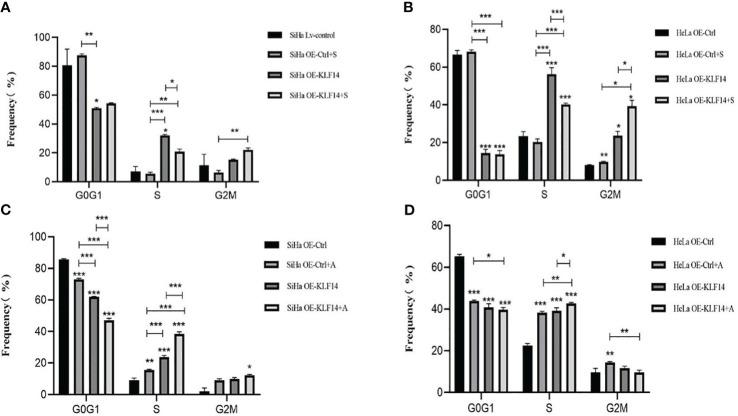
KLF14 induces S-phase arrest in CC cells by activating the JNK pathway. OE-KLF14 group and OE-Ctrl group were treated with 20μmol/l SP600125**(A, B)** or 0.125μmol/l anisomycin **(C, D)** to inhibit/promote JNK pathway activation. The proportion of cell cycle in each group was detected by flow cytometry and statistically analyzed (*P < 0.05, **P < 0.01, ***P < 0.001).

After SP600125 treatment, the ratio of cells in the S phase in the OE-KLF14+S group was lower than in the control group (*P*_SiHa_=0.020, *P*_HeLa_<0.001) (**Figures 6A, B**). We noted that inhibition of JNK-pathway activation reversed KLF14-induced S-phase arrest and that after anisomycin treatment the ratio of cells in the S phase in both the OE-KLF14+A and OE-Ctrl+A groups was elevated relative to their respective control group (*P*_SiHa_<0.001, *P*_HeLa_=0.014; and *P*_SiHa_=0.001, *P*_HeLa_<0.001; respectively) (**Figures 6C, D**). These results showed that KLF14 induces S-phase arrest of cervical cancer cells by activating the JNK-signaling pathway.

**Figure 7:**
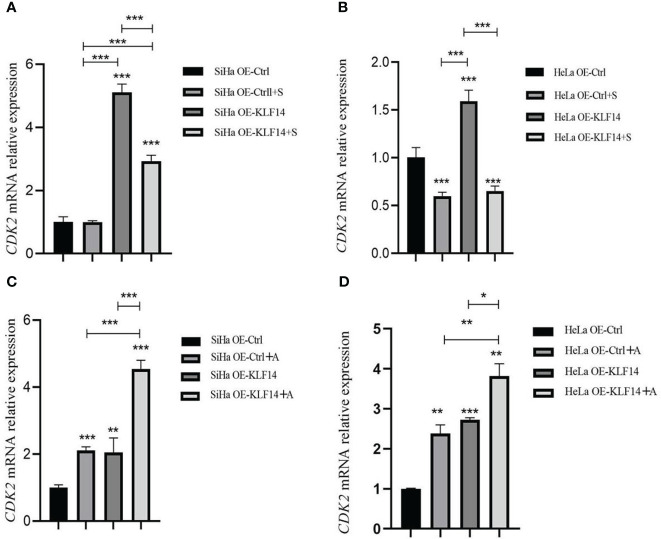
KLF14 promoted CDK2 mRNA expression in CC cells by activating the JNK pathway. OE-KLF14 group and OE-Ctrl group were treated with 20μmol/l SP600125**(A, B)** or 0.125μmol/l anisomycin **(C, D)** to inhibit/promote JNK pathway activation. The mRNA expression of CDK2 in each group was detected by qRT-PCR. (*P < 0.05, **P < 0.01, ***P < 0.001).

After SP600125 treatment, the *CDK2* mRNA expression level in the OE-KLF14+S group was lower than its control group (all *P*<0.001) (**Figures 7A, B**), indicating that inhibition of JNK-pathway activation reversed KLF14-induced *CDK2* mRNA expression in cervical cancer cells. After anisomycin treatment, the *CDK2* mRNA expression level of the OE-KLF14+A group was increased relative to its control group (*P*_SiHa_<0.001, *P*_HeLa_=0.030) (**Figures 7C, D**). This showed that KLF14 increases *CDK2* mRNA expression by activating the JNK pathway in cervical cancer cells.

**Figure 8:**
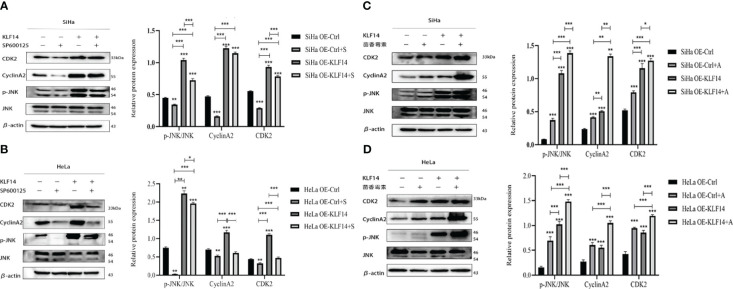
KLF14 promotes the expression of CDK2 and CyclinA2 proteins in CC cells by activating the JNK pathway. OE-KLF14 group and OE-Ctrl group were treated with 20μmol/l SP600125**(A, B)** or 0.125μmol/l anisomycin **(C, D)** to inhibit/promote JNK pathway activation. Expression of JNK, p-JNK, CDK2, and CyclinA2 by Western blot assay. (*P < 0.05, **P < 0.01, ***P < 0.001).

After SP600125 treatment, the p-JNK/JNK ratio and the protein expression of CDK2 and CyclinA2 in the OE-KLF14+S group was lower than in its control group (*P*_SiHa_<0.001, *P*_HeLa_=0.049; *P*_SiHa_ *<*0.001, *P*_HeLa_ *<*0.001; *P*_SiHa_=0.006, *P*_HeLa_<0.001) (**Figures 8A, B**). This demonstrated that inhibition of JNK-pathway activation reversed KLF14-induced CDK2 and CyclinA2 protein expression in cervical cancer cells. After anisomycin treatment, the p-JNK/JNK ratio and the protein expression of CDK2 and CyclinA2 in the OE-KLF14+S group was elevated relative to its control group (*P*_SiHa_ *<*0.001, *P*_HeLa_ *<*0.001; *P*_SiHa_ *=*0.049, *P*_HeLa_<0.001; *P*_SiHa_=0.001, *P*_HeLa_<0.001) (**Figures 8C, D**). This result revealed that promoting JNK-pathway activation by KLF14 promoted CDK2 and CyclinA2 protein expression in cervical cancer cells.

The above experiments revealed that KLF14 activated the JNK-signaling pathway to induce S-phase arrest and promote CDK2 and CyclinA2 expression in cervical cancer cells.

### 3.6 KLF14 restrains the growth of cervical cancer xenografts *in vivo*

**Figure 9:**
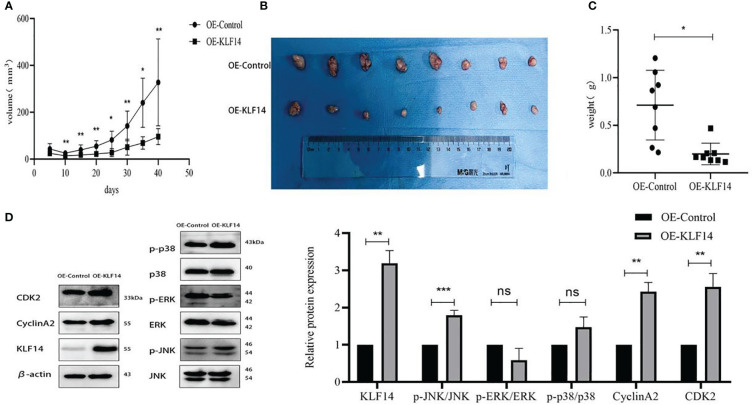
KLF14 inhibited CC cell proliferation *in vivo*. **(A)** The effects of KLF14 on proliferation and related protein expression *in vivo* were investigated in Subcutaneous xenograft studies. SiHa OE-Ctrl and SiHa OE-KLF14 were injected subcutaneously into the left and right axillae, respectively. The results showed that tumour growth was faster in the OE-Ctrl group than in the OE-KLF14 group. **(B)** Forty days later, the nude mice were euthanized under anesthesia, and the size of subcutaneous tumours in the OE-KLF14 group was smaller than that in the OE-Ctrl group. **(C)** The weight of the subcutaneous tumor in the OE-KLF14 group was lower than that in the OE-Ctrl group. **(D)** Expression of KLF14, p-JNK/JNK, CDK2, and CyclinA2 by western blot assay was upregulated in the OE-KLF14 group compared with the OE-Ctrl group in tumour tissues. (*P < 0.05, **P < 0.01, ***P < 0.001, ns: no significance).

We constructed a subcutaneous tumour-bearing nude mouse model and plotted its tumour growth curve (**Figure 9A**). *S-W* normality testing revealed that tumour volumes in the experimental groups on Day 25 (*P*=0.003) and Day 35 (*P*=0.046) were not normally distributed while the remaining tumour volumes followed a normal distribution. When we compared tumour volumes between the OE-KLF14 group and its control group, we ascertained no difference on Day 5 (*P*=0.076) but noted a significant difference on Days 10 (*P*=0.003), 15 (*P*=0.004), 20 (*P*=0.006), 25 (*P*=0.012), 30 (*P*=0.002), 35 (*P*=0.012), and 40 (*P*=0.005). On Day 40 of model construction, the nude mice were euthanized under anesthesia. The tumours that had formed under the skin were removed and photographed (**Figure 9B**). Our results showed that tumours in the experimental group weighed less than in the control group (*P*=0.012) (**Figure 9C**) and that KLF14 inhibited the proliferation of cervical cancer cells *in vivo*. For further verification, we explored the expression of KLF14 and other related molecules in tumour tissues. According to the Western blot, the expression of KLF14, CDK2, CyclinA2, and *p*-JNK/JNK were upregulated (*P*_KLF14 =_ 0.008*, P*_CDK2 =_ 0.002, *P*_CyclinA2 =_ 0.009, *P_p_*_-JNK/JNK_<0.001), while *p*-ERK/ERK and *p*-P38/P38 did not differ (*P_p_*_-ERK/ERK_=0.154, *P_p_*_-p38/p38 =_ 0.097) in the OE-KLF14 group compared with the OE-Ctrl group (**Figure 9D**).

## 4 Discussion

Cervical cancer is an invasive type of cancer and the fourth most common cancer in women worldwide (30). Most patients are at a clinically advanced stage when they are diagnosed, and our understanding of the potential molecular and genetic mechanisms underlying cervical cancer is limited (31). For this study, we primarily sought molecules associated with cervical cancer tumorigenesis and progression and provided novel targets for cervical cancer treatment.

SP/KLF proteins are members of a transcription factor family that is characterized by three highly conserved zinc fingers that interact with DNA. Investigators have demonstrated the presence of SP/KLF transcription factors in many tissues and that they regulate cellular proliferation, differentiation, apoptosis, and tumorigenesis (32). As a member of this family, KLF14 occupies unique positions in signaling pathways, proliferation, and cellular differentiation. Studies on KLF14 have in recent years gradually expanded to include tumorigenesis and immune regulation (12, 33). KLF14 binds to the miR-1283 promotor and enhances expression to inhibit the progression of HER2+ breast cancer (34). KLF14 suppressed breast cancer cell invasion and M2 macrophage polarization through modulating SOCS3/RhoA/Rock/STAT3 signaling (16).

Cell-cycle progression constitutes the principal mechanism underlying the regulation of cellular growth (35, 36), and many cytotoxic agents and DNA-damaging agents arrest the cell cycle at the G0/G1, S, or G2/M phases before inducing cell death (37, 38). The cell cycle is regulated by cyclin-dependent kinases (CDKs) and their cell-cycle protein partners. Among CDKs that regulate cell cycle progression, CDK2 is a critical kinase that regulates S-phase entry and progression and binds to CCNA2 to accomplish its effects (39). In HeLa cells, KLF14 overexpression caused a threefold increase of cells in the G2/M phase. KLF14 leads to an increase in the proportion of cells in M-phase. KLF14 remarkably increases the levels of cyclin B1 and phosphor-histone H3 (two mitosis markers). Whereas KLF14-ZF2(deletion of zinc finger 2 in KLF14) has no such effects. Co-expression of PIK4 only partially blocked KLF14 overexpression-induced cell cycle arrest at G2/M-phase, suggesting that mechanisms other than PIK4 inhibition also contribute to KLF14 overexpression-induced cell cycle arrest (20). Our group previously employed CCK-8 and colony-formation assays, and subcutaneous tumorigenesis experiments in nude mice to show that KLF14 inhibited the proliferation of cervical cancer cells. We used flow cytometry to demonstrate that KLF14 promoted apoptosis of cervical cancer cells, and in our mechanistic exploration, we found that it targets *ITGB1* to regulate downstream *PI3K*/*AKT* signaling—thus promoting apoptosis (18). The results of the present study further showed that KLF14 induces S-phase arrest in cervical cancer and activates the JNK pathway to promote the expression of CDK2 and CCNA2 in cervical cancer cells, with its zinc fingers participating in the induction of S-phase arrest.

JNK, ERK, and p38 proteins in the MAPK-signaling pathway are major mediators of the cellular responses to extracellular signals and play critical roles in tumor survival, proliferation, and cell-cycle progression. JNK pathways are activated by a variety of extracellular stimuli (e.g., cytokines, pathogens, morphogenic factors, hormones) as well as intracellular stimuli (e.g., oxidative stress, DNA damage). About JNK nucleo-cytoplasmic trafficking, JNK kinases can change their subcellular localization upon pathway activation via either preferential nuclear localization or enhanced nuclear retention. A study by Fujimoto revealed that the JNK and ERK-signaling pathways were crucial in NFD(naphtho[1,2-b]furan-4,5-dione)-induced S-phase arrest and apoptosis in MDA-MB-231 cells (39). KLF14 and the MAPK-signaling pathway are also closely correlated with the expression of inflammatory factors in oxidative adaptation in prostate cancer and atherosclerosis (24, 25). As a transcription factor located in the nucleus, KLF14 may affect the activation of the JNK pathway through intracellular stimulation. The results suggest that KLF14 promoted JNK-pathway activation to induce S-phase arrest and promote the expression of CDK2 and CCNA2 in cervical cancer cells. However, the exact mechanism by which KL14 directly or indirectly regulates JNK activity needs to be further explored (40).

In summary, this study revealed that KLF14 inhibited the proliferation of cervical cancer cells *in vivo*, and we for the first time determined that KLF14 induced S-phase arrest in cervical cancer cells and that the action was related to its zinc-finger structure. KLF14 also activated the JNK pathway to induce S-phase arrest and promoted the expression of CDK2 and CCNA2. We therefore demonstrated a role for KLF14 in regulating the cell cycle in cervical cancer and uncovered its underlying mechanism of action. This study, thus, provided novel concepts for genetic engineering studies, and we created a theoretical basis for the further development of genetically engineered drugs to treat cervical cancer. We also validated several important molecular sites where KLF14 achieved its effects. The presence of zinc fingers in KLF14 promotes S-phase arrest in cervical cancer cells, and we postulate that this aspect will generate a basis for targeted therapy in cervical cancer and a theoretical platform for the development of cervical cancer biopharmaceuticals. As many signaling pathways participate in the cell cycle and since the regulatory network of KLF14 is complex, we only ascertained a correlation among KLF14, the JNK pathway, and cell cycle-related proteins. However, the specific molecular targeting mechanisms remain unclear. In future studies, our research group will further examine the role of KLF14 and discern its underlying mechanisms of action in various oncological behaviors in cervical cancer, thereby providing a novel foundation for the development of therapeutic drug targets of KLF14.

## Data availability statement

The original contributions presented in the study are included in the article/supplementary materials, further inquiries can be directed to the corresponding author/s.

## Ethics statement

The animal study was approved by the Animal Ethics Committee of the First Affiliated Hospital of Shandong First Medical University, with the approval number of SYDWLS (2020) 016. The study was conducted in accordance with the local legislation and institutional requirements.

## Author contributions

YD: Writing – original draft. HY: Writing – original draft. ML: Methodology, Writing – review & editing. LC: Writing – original draft, Writing – review & editing.

## References

[1] Siegel RL Miller KD Fuchs HE Jemal A. Cancer statistics, 2021. CA Cancer J Clin (2021) 71(1):7–33. doi: 10.3322/caac.21654 33433946

[2] Xia C Dong X Li H Cao M Sun D He S. Cancer statistics in China and United States, 2022: profiles, trends, and determinants. Chin Med J (2022) 135(5):584–90. doi: 10.1097/CM9.0000000000002108 PMC8920425 35143424

[3] Wang R Pan W Jin L Huang W Li Y Wu D. Human papillomavirus vaccine against cervical cancer: Opportunity and challenge. Cancer Lett (2020) 471:88–102. doi: 10.1016/j.canlet.2019.11.039 31812696

[4] Scarth JA Patterson MR Morgan EL Macdonald A. The human papillomavirus oncoproteins: a review of the host pathways targeted on the road to transformation. J Gen Virol (2021) 102(3):001540. doi: 10.1099/jgv.0.001540 33427604 PMC8148304

[5] Burmeister CA Khan SF Schäfer G Mbatani N Adams T Moodley J. Cervical cancer therapies: Current challenges and future perspectives. Tumour Virus Res (2022) 13:200238. doi: 10.1016/j.tvr.2022.200238 35460940 PMC9062473

[6] Gupta S Kumar P Das BC. HPV: Molecular pathways and targets. Curr problems Cancer (2018) 42(2):161–74. doi: 10.1016/j.currproblcancer.2018.03.003 29706467

[7] Bhattacharjee R Das SS Biswal SS Nath A Das D Basu A. Mechanistic role of HPV-associated early proteins in cervical cancer: Molecular pathways and targeted therapeutic strategies. Crit Rev oncology/hematology (2022) 174:103675. doi: 10.1016/j.critrevonc.2022.103675 35381343

[8] Manzo-Merino J Del-Toro-Arreola S Rocha-Zavaleta L Peralta-Zaragoza Ó Jiménez-Lima R Madrid-Marina V. IMMUNOLOGY OF CERVICAL CANCER. Rev investigacion clinica; organo del Hosp Enfermedades la Nutricion (2020) 72(4):188–97. doi: 10.24875/RIC.20000057 33064686

[9] Evan GI Vousden KH. Proliferation, cell cycle and apoptosis in cancer. Nature (2001) 411(6835):342–8. doi: 10.1038/35077213 11357141

[10] Liu J Peng Y Wei W. Cell cycle on the crossroad of tumorigenesis and cancer therapy. Trends Cell Biol (2022) 32(1):30–44. doi: 10.1016/j.tcb.2021.07.001 34304958 PMC8688170

[11] Bureau C Hanoun N Torrisani J Vinel J P Buscail L Cordelier P. Expression and function of kruppel like-factors (KLF) in carcinogenesis. Curr Genomics (2009) 10(5):353–60. doi: 10.2174/138920209788921010 PMC2729999 20119532

[12] Chen X Shi W Zhang H. The role of KLF14 in multiple disease processes. BioFactors (Oxford England) (2020) 46(2):276–82. doi: 10.1002/biof.1612 31925990

[13] Wu G Yuan S Chen Z Chen G Fan Q Dong H. The KLF14 transcription factor regulates glycolysis by downregulating LDHB in colorectal cancer. Int J Biol Sci (2019) 15(3):628–35. doi: 10.7150/ijbs.30652 PMC6367579 30745849

[14] Zahra K Shabbir M Badshah Y Trembley J H Badar Z Khan K. Determining KLF14 tertiary structure and diagnostic significance in brain cancer progression. Sci Rep (2022) 12(1):8039. doi: 10.1038/s41598-022-12072-0 35577881 PMC9110742

[15] Li Z Yao H Wang S Li G Gu X. CircTADA2A suppresses the progression of colorectal cancer *via* miR-374a-3p/KLF14 axis. J Exp Clin Cancer Res CR (2020) 39(1):160. doi: 10.1186/s13046-020-01642-7 32799891 PMC7429896

[16] Chu J Hu XC Li CC Li TY Fan HW Jiang GQ. KLF14 alleviated breast cancer invasion and M2 macrophages polarization through modulating SOCS3/RhoA/Rock/STAT3 signaling. Cell signalling (2022) 92:110242. doi: 10.1016/j.cellsig.2022.110242 34998931

[17] Wang YG Liu J Shi M Chen F X. LncRNA DGCR5 represses the development of hepatocellular carcinoma by targeting the miR-346/KLF14 axis. J Cell Physiol (2018) 234(1):572–80. doi: 10.1002/jcp.26779 30216442

[18] Lyu X Ding X Ye H Guo R Wu M Cao L. KLF14 targets ITGB1 to inhibit the progression of cervical cancer *via* the PI3K/AKT signalling pathway. Discover Oncol (2022) 13(1):30. doi: 10.1007/s12672-022-00494-1 PMC9108130 35570248

[19] Zhou J Lin J Zhang H Zhu F Xie R. LncRNA HAND2-AS1 sponging miR-1275 suppresses colorectal cancer progression by upregulating KLF14. Biochem Biophys Res Commun (2018) 503(3):1848–53. doi: 10.1016/j.bbrc.2018.07.125 30078677

[20] Fan G Sun L Shan P Zhang X Huan J Zhang X. Loss of KLF14 triggers centrosome amplification and tumorigenesis. Nat Commun (2015) 6:8450. doi: 10.1038/ncomms9450 26439168 PMC4600754

[21] Teng JA Wu SG Chen JX Li Q Peng F Zhu Z. The activation of ERK1/2 and JNK MAPK signaling by insulin/IGF-1 is responsible for the development of colon cancer with type 2 diabetes mellitus. PloS One (2016) 11(2):e0149822. doi: 10.1371/journal.pone.0149822 26901856 PMC4763097

[22] Bogoyevitch MA Boehm I Oakley A Ketterman AJ Barr RK. Targeting the JNK MAPK cascade for inhibition: basic science and therapeutic potential. Biochim Biophys Acta (2004) 1697(1-2):89–101. doi: 10.1016/j.bbapap.2003.11.016 15023353

[23] Wagner EF Nebreda AR. Signal integration by JNK and p38 MAPK pathways in cancer development. Nat Rev Cancer (2009) 9(8):537–49. doi: 10.1038/nrc2694 19629069

[24] Luo XH Liu JZ Wang B Men QL Ju YQ Yin FY. KLF14 potentiates oxidative adaptation *via* modulating HO-1 signaling in castrate-resistant prostate cancer. Endocrine-related Cancer (2019) 26(1):181–95. doi: 10.1530/ERC-18-0383 30400002

[25] Wei X Yang R Wang C Jian X Li L Liu H. A novel role for the Krüppel-like factor 14 on macrophage inflammatory response and atherosclerosis development. Cardiovasc Pathol Off J Soc Cardiovasc Pathol (2017) 27:1–8. doi: 10.1016/j.carpath.2016.11.003 27923151

[26] Hsieh WT Lin HY Chen JH Lin WC Kuo YH Wood WG. Latex of Euphorbia antiquorum-induced S-phase arrest *via* active ATM kinase and MAPK pathways in human cervical cancer HeLa cells. Environ Toxicol (2015) 30(10):1205–15. doi: 10.1002/tox.21992 24706497

[27] Zhang T Li B Feng Q Xu Z Huang C Wu H. DCZ0801, a novel compound, induces cell apoptosis and cell cycle arrest *via* MAPK pathway in multiple myeloma. Acta Biochim Biophys Sin (2019) 51(5):517–23. doi: 10.1093/abbs/gmz033 30947332

[28] Wang D Sun Q Wu J Wang W Yao G Li T. A new Prenylated Flavonoid induces G0/G1 arrest and apoptosis through p38/JNK MAPK pathways in Human Hepatocellular Carcinoma cells. Sci Rep (2017) 7(1):5736. doi: 10.1038/s41598-017-05955-0 28720813 PMC5515844

[29] Zhang T Liu WD Saunee NA Breslin MB Lan MS. Zinc finger transcription factor INSM1 interrupts cyclin D1 and CDK4 binding and induces cell cycle arrest. J Biol Chem (2009) 284(9):5574–81. doi: 10.1074/jbc.M808843200 PMC2645817 19124461

[30] Buskwofie A David-West G Clare CA. A review of cervical cancer: incidence and disparities. J Natl Med Assoc (2020) 112(2):229–32. doi: 10.1016/j.jnma.2020.03.002 32278478

[31] Olusola P Banerjee HN Philley JV Dasgupta S. Human papilloma virus-associated cervical cancer and health disparities. Cells (2019) 8(6):622. doi: 10.3390/cells8060622 31234354 PMC6628030

[32] Orzechowska-Licari EJ LaComb JF Mojumdar A Bialkowska AB. SP and KLF transcription factors in cancer metabolism. Int J Mol Sci (2022) 23(17):9956. doi: 10.3390/ijms23179956 36077352 PMC9456310

[33] Yang Q Civelek M. Transcription factor KLF14 and metabolic syndrome. Front Cardiovasc Med (2020) 7:91. doi: 10.3389/fcvm.2020.00091 32548128 PMC7274157

[34] Chen XZ He WX Luo RG Xia GJ Zhong JX Chen QJ. KLF14/miR-1283/TFAP2C axis inhibits HER2-positive breast cancer progression via declining tumor cell proliferation. Mol Carcinogenesis (2023) 62(4):532–45. doi: 10.1002/mc.23505 36752341

[35] Gamet-Payrastre L Li P Lumeau S Cassar G Dupont MA Chevolleau S. Sulforaphane, a naturally occurring isothiocyanate, induces cell cycle arrest and apoptosis in HT29 human colon cancer cells. Cancer Res (2000) 60(5):1426–33. 10728709

[36] Murray AW. Recycling the cell cycle: cyclins revisited. Cell (2004) 116(2):221–34. doi: 10.1016/S0092-8674(03)01080-8 14744433

[37] Orren DK Petersen LN Bohr VA. Persistent DNA damage inhibits S-phase and G2 progression, and results in apoptosis. Mol Biol Cell (1997) 8(6):1129–42. doi: 10.1091/mbc.8.6.1129 PMC305719 9201721

[38] Fujimoto K Hosotani R Doi R Wada M Lee J U Koshiba T. Induction of cell-cycle arrest and apoptosis by a novel retinobenzoic-acid derivative, TAC-101, in human pancreatic-cancer cells. Int J Cancer (1999) 81(4):637–44. doi: 10.1002/(SICI)1097-0215(19990517)81:4<637::AID-IJC21>3.0.CO;2-4 10225456

[39] Lin KL Su JC Chien CM Tseng CH Chen YL Chang LS. Naphtho[1,2-b]furan-4,5-dione induces apoptosis and S-phase arrest of MDA-MB-231 cells through JNK and ERK signaling activation. Toxicol Vitro an Int J published Assoc BIBRA (2010) 24(1):61–70. doi: 10.1016/j.tiv.2009.09.002 19747539

[40] Zeke A Misheva M Reményi A Bogoyevitch MA. JNK signaling: regulation and functions based on complex protein-protein partnerships. Microbiol Mol Biol Rev MMBR (2016) 80(3):793–835. doi: 10.1128/MMBR.00043-14 27466283 PMC4981676

